# Reproducibility: Reliability and Agreement Parameters of the Revised Short McGill Pain Questionnaire Version-2 for use in Patients with Musculoskeletal Shoulder Pain

**DOI:** 10.1080/24740527.2020.1712653

**Published:** 2020-12-30

**Authors:** Samuel U. Jumbo, Joy C. MacDermid, Tara L. Packham, George S. Athwal, Kenneth J. Faber

**Affiliations:** a Faculty of Health and Rehabilitation Sciences, Elborn College, Western University, London, Ontario, Canada; b School of Rehabilitation Science, McMaster University, Hamilton, Ontario, Canada; c Roth McFarlane Hand and Upper Limb Centre, St. Joseph’s Hospital, London, Ontario, Canada

**Keywords:** reproducibility, reliability, agreement, McGill Pain Questionnaire, shoulder pain, musculoskeletal, patient-reported outcomes

## Abstract

**Background**: The Revised Short-Form McGill Pain Questionnaire Version-2 (SF-MPQ-2) is a multidimensional outcome measure designed to evaluate neuropathic and nonneuropathic pain. A recent systematic review found insufficient psychometric data with respect to musculoskeletal health conditions.

**Aims**: The aim of this study was to describe the reproducibility (reliability and agreement) and internal consistency of the SF-MPQ-2 for use among patients with musculoskeletal shoulder pain.

**Methods**: Eligible patients with shoulder pain from musculoskeletal (MSK) sources completed the SF-MPQ-2 at baseline (*n* = 195), and a subset did so again after 3 to 7 days (*n* = 48) if their response to the global rating of change scale remained unchanged. Cronbach’s alpha (α) and intraclass correlation coefficient (ICC[2,1]) were calculated. Standard error of measurement (SEM), group and individual minimal detectable change (MDC90), and Bland-Altman plots were used to assess agreement.

**Results**: Cronbach’s α ranged from 0.83 to 0.95, suggesting very satisfactory internal consistency across the SF-MPQ-2 domains. Excellent ICC(2,1) scores were found in support of the total (0.95) and continuous (0.92) subscales; the remaining subscales displayed good ICC(2,1) scores (0.78–0.88). Bland-Altman analysis revealed no systematic bias between the test and retest scores (mean difference = 0.13 to 0.19). Though the best agreement coefficients were seen on the total scale (SEM = 0.5; MDC90 = 1.2, MDC90_group_ = 0.3), they were acceptable for the SF-MPQ-2 subscales (SEM: range, 0.7–1; MDC90: range, 1.7–2.3; MDC90_group_: range, 0.4–0.5).

**Conclusions**: The SF-MPQ-2 provides good to excellent test–retest reliability for multidimensional pain assessment among patients with musculoskeletal shoulder pain conditions.

## RÉSUMÉ

**Contexte**: Le Questionnaire abrégé révisé de McGill sur la douleur, version 2 (SF-MPQ-2) est une mesure multidimensionnelle des résultats conçue pour évaluer la douleur neuropathique et non neuropathique. Une revue systématique récente a révélé l'insuffisance des données psychométriques relatives aux troubles musculo-squelettiques.

**Objectifs**: décrire la reproductibilité (fiabilité et concordance) et la cohérence interne du SF-MPQ-2 pour une utilisation chez les patients souffrant de douleurs musculo-squelettiques à l'épaule.

**Méthodes**: Les patients admissibles souffrant de douleurs à l'épaule provenant de sources musculo-squelettiques ont répondu au SF-MPQ-2 : au départ (n = 195), et un sous-ensemble l'a fait à nouveau après une période de 3 à 7 jours (n  =  48), si leur réponse pour l'échelle de l’évaluation globale du changement était restée inchangée. Le coefficient alpha de Cronbach (α) et le coefficient de corrélation intra-classe (CCI2, 1) ont été calculés. L'erreur-type de mesure (ETM), le changement minimal détectable (CMD90) pour le groupe et pour l'individu, et les graphiques de Bland-Altman (BA) ont été utilisés pour évaluer la concordance.

**Résultats**: L’alpha de Cronbach se situait entre 0,83 à 0,95, ce qui indique une cohérence interne très satisfaisante dans les domaines du SF-MPQ-2. D'excellents scores CCI2, 1 ont été trouvés à l'appui des sous-échelles totale (0,95) et continue (0,92) ; les autres sous-échelles ont affiché de bons scores CCI2, 1 (0,78 à 0,88). L'analyse de Bland-Altman n'a révélé aucun biais systématique entre les résultats du test et ceux du retest (différence moyenne = 0,13 à 0,19). Bien que les meilleurs coefficients de concordance aient été observés sur l'échelle totale (ETM  =  0,5 ; CMD90 = 1,2 et groupe CMD90 = 0,3), ils étaient acceptables pour les deux sous-échelles du SF-MPQ-2 (ETM :   entre 0,7 et 1 ; ETM90 :   entre 1,7 et 2,3 ; groupe CMD90 :   entre 0,4 et 0,5).

**Conclusions**: Le SF-MPQ-2 offre une fiabilité test-retest allant de bonne à excellente pour l'évaluation multidimensionnelle de la douleur chez les patients souffrant de douleurs musculo-squelettiques de l'épaule.

